# Brief summary of French guidelines for the prevention, diagnosis and treatment of hospital-acquired pneumonia in ICU

**DOI:** 10.1186/s13613-018-0444-0

**Published:** 2018-11-03

**Authors:** Marc Leone, Lila Bouadma, Bélaïd Bouhemad, Olivier Brissaud, Stéphane Dauger, Sébastien Gibot, Sami Hraiech, Boris Jung, Eric Kipnis, Yoann Launey, Charles-Edouard Luyt, Dimitri Margetis, Fabrice Michel, Djamel Mokart, Philippe Montravers, Antoine Monsel, Saad Nseir, Jérôme Pugin, Antoine Roquilly, Lionel Velly, Jean-Ralph Zahar, Rémi Bruyère, Gérald Chanques, ADARPEF, Fabrice Michel, GFRUP, Stephane Dauger, Olivier Brissaud

**Affiliations:** 1 Service d’Anesthésie et de Réanimation, Aix-Marseille Universite Hopital Nord, chemin des Bourrely, 13015 Marseille, France; 2 Service de Réanimation Médicale, Hopital Bichat - Claude-Bernard, AP-HP, Paris, France; 3 Service d’Anesthésie et Réanimation, Centre Hospitalier Universitaire de Dijon, Paris, France; 4 Unité de Réanimation Pédiatrique, Hôpital Pellegrin, Centre Hospitalier Universitaire de Bordeaux, Bordeaux, France; 5 Service de Réanimation Pédiatrique, Hopital Universitaire Robert-Debre, Paris, France; 6 Service de Réanimation Médicale, CHU de Nancy, Vandoeuvre-les-Nancy, France; 7 Service de Réanimation des Détresses Respiratoires et des Infections Sévères, Aix-Marseille Universite, Hopital Nord, Marseille, France; 8 Service d’Anesthésie et Réanimation, CHU de Montpellier, Montpellier, France; 9 Service d’Anesthésie et Réanimation, CHU de Lille, Lille, France; 10 Service d’Anesthésie et Réanimation, Centre Hospitalier Universitaire de Rennes, Rennes, France; 11 Institut de Cardiologie, Service de Réanimation Médicale, Hopital Universitaire Pitie Salpetriere, AP-HP, Paris, France; 12 Service de Réanimation Médicale - Hôpital Saint-Antoine, Paris, France; 13 Service d’Anesthésie et Réanimation, Hopital La timone, Assistance Publique Hopitaux de Marseille, Marseille, France; 14 Service de Réanimation, Institut Paoli-Calmettes, Marseille, France; 15 Département d’Anesthésie Réanimation, CHU Bichat - Claude-Bernard, AP-HP, Paris, France; 16 Département d’Anesthésie et Réanimation, Université Pierre et Marie Curie, Paris, France; 17 Centre de Soins Intensifs, Service de Réanimation, Centre Hospitalier Regional Universitaire de Lille, Lille, France; 18 Service de Soins Intensifs, Hopitaux Universitaires de Geneve, Geneve, Switzerland; 19 Service d’Anesthésie et Réanimation, CHU de Nantes, Nantes, France; 20 Département d’Anesthésie et Réanimation, Hopital de la Timone, AP-HM, Paris, France; 21 Département de Microbiologie Clinique, Hopital Avicenne, APHP, Paris, France; 22 Service de Réanimation, Centre Hospitalier de Bourg-en-Bresse, Bourg-en-Bresse, France; 23 Département d’Anesthésie Réanimation, Centre Hospitalier Regional Universitaire de Montpellier, Montpellier, France

## Abstract

**Background:**

The French Society of Anaesthesia and Intensive Care Medicine and the French Society of Intensive Care edited guidelines focused on hospital-acquired pneumonia (HAP) in intensive care unit. The goal of 16 French-speaking experts was to produce a framework enabling an easier decision-making process for intensivists.

**Results:**

The guidelines were related to 3 specific areas related to HAP (prevention, diagnosis and treatment) in 4 identified patient populations (COPD, neutropenia, post-operative and paediatric). The literature analysis and the formulation of the guidelines were conducted according to the Grade of Recommendation Assessment, Development and Evaluation methodology. An extensive literature research over the last 10 years was conducted based on publications indexed in PubMed™ and Cochrane™ databases.

**Conclusions:**

HAP should be prevented by a standardised multimodal approach and the use of selective digestive decontamination in units where multidrug-resistant bacteria prevalence was below 20%. Diagnosis relies on clinical assessment and microbiological findings. Monotherapy, in the absence of risk factors for multidrug-resistant bacteria, non-fermenting Gram-negative bacilli and/or increased mortality (septic shock, organ failure), is strongly recommended. After microbiological documentation, it is recommended to reduce the spectrum and to prefer monotherapy for the antibiotic therapy of HAP, including for non-fermenting Gram-negative bacilli.

## Introduction

**Fig. 1:**
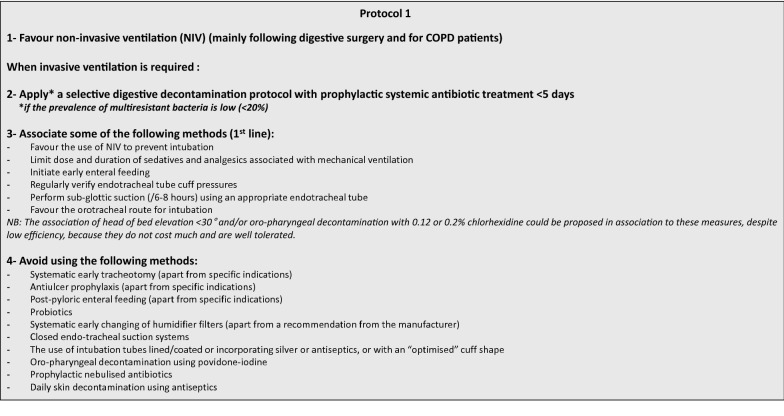
Multimodal healthcare associated pneumonia prevention protocol (expert opinion)

Hospital-acquired pneumonia (HAP) is the most common infection in the intensive care unit (ICU) [1]. In the ICU, HAP is associated with a mortality rate of 20% and with increased duration of mechanical ventilation and ICU and hospital length-of-stay [2, 3]. The criteria to diagnose pneumonia are shown in Table 1 (Fig. 1).

**Table 1:** Criteria for defining pneumonia

Radiological signs
Two successive chest radiographs showing new or progressive lung infiltrates In the absence of medical history of underlying heart or lung disease, a single chest radiograph is enough
And at least one of the following signs
Body temperature > 38,3 °C without any other cause Leucocytes < 4000/mm^3^ or ≥ 12,000/mm^3^
And at least two of the following signs
Purulent sputum Cough or dyspnoea Declining oxygenation or increased oxygen requirement or need for respiratory assistance

## Method

**Fig. 2:**
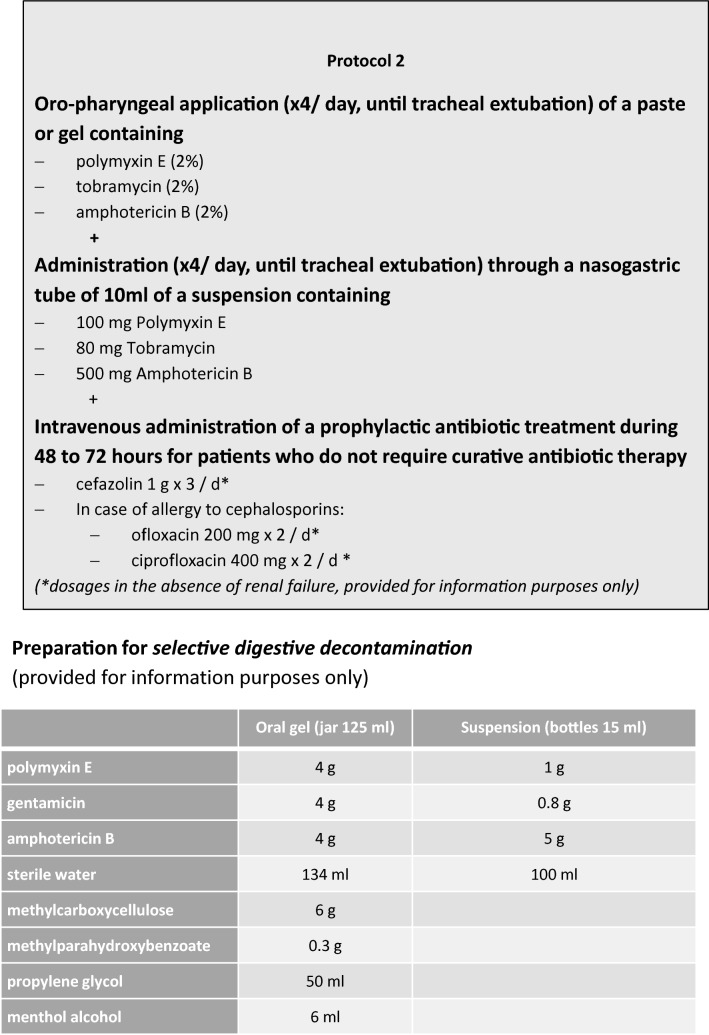
Selective digestive decontamination protocol (expert opinion)

Sixteen French-speaking experts produce guidelines in three specific areas related to HAP: prevention, diagnosis and treatment as well as the specificities pertaining to different identified patient populations (COPD, neutropenia, post-operative and paediatric). The schedule of the group was defined upstream (Table 2) (Fig. 2).

**Table 2:** Guideline timeline

5 December 2016	Start-up meeting
6 March 2017	Vote: first round
13 March 2017	Post-vote deliberation meeting
1 April 2017	Vote: second round
16 April 2017	Amendment of two guidelines
28 April 2017	Vote of the two amended guidelines
10 May 2017	Guideline finalisation meeting

**Fig. 3:**
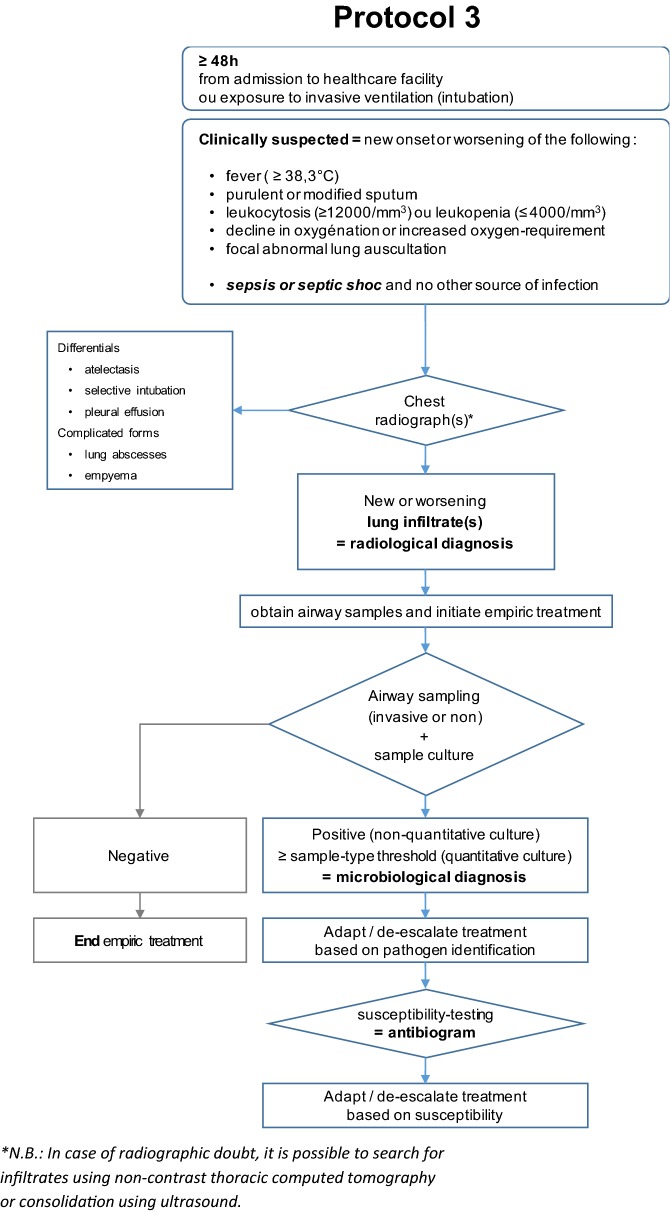
Diagnostic procedure (expert opinion)

The questions were formulated according to the PICO (Patient, Intervention, Comparison, Outcome) format. The formulation of the guidelines was conducted according to the GRADE methodology (Grade of Recommendation Assessment, Development and Evaluation) [4, 5]. In the absence of supporting literature, a question could be addressed by a recommendation under the form of an expert opinion (“the experts suggest that…”) (Fig. 3).

**Fig. 4:**
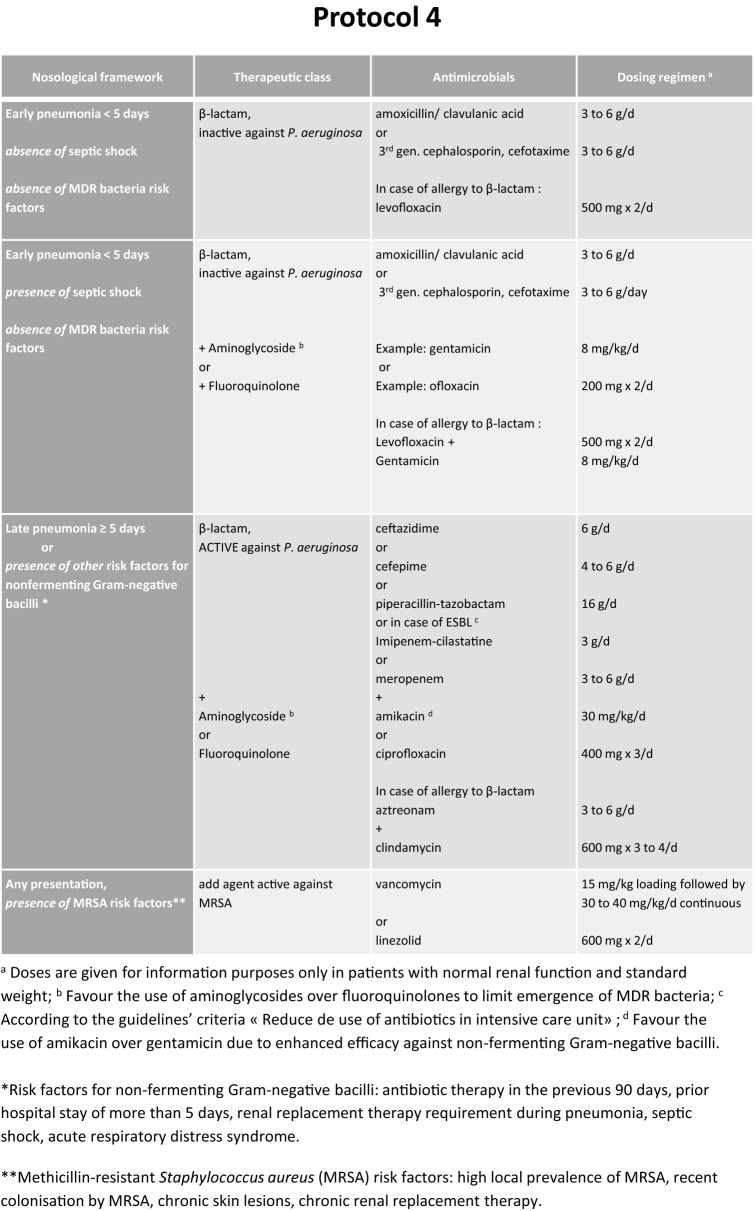
Treatment options (expert opinion)

These guidelines with their arguments were published in the journal Anaesthesia Critical Care and Pain Medicine [6] (Fig. 4).

**First area, PREVENTION** Which HAP prevention approaches decrease morbidity and mortality in ICU patients?

R1.1 We recommend using a standardised multimodal HAP prevention approach in order to decrease ICU patient morbidity (Grade 1+).
R1.1 Paediatrics We suggest using a standardised multimodal approach aiming at preventing HAP in order to decrease paediatric ICU patient morbidity (Grade 2+).
R1.2 In units where multidrug-resistant bacteria prevalence is low (< 20%), we suggest applying routine selective digestive decontamination using a topical antiseptic administered enterally and a maximal 5-day course of systemic prophylactic antibiotic to decrease mortality (Grade 2+).
R1.3 Within a standardised multimodal HAP prevention approach, we suggest combining some of the following methods to decrease ICU patient morbidity:
  Promote the use of non-invasive ventilation to avoid tracheal intubation (mainly in post-operative digestive surgery patients and in patients with COPD),
  Favour orotracheal over nasotracheal intubation when required
  Limit dose and duration of sedatives and analgesics (promote their use guided by sedation/pain/agitation scales, and/or daily interruptions),
  Initiate early enteral feeding (within the first 48 h of ICU admission),
  Regularly verify endotracheal tube cuff pressure,
  Perform sub-glottic suction (every 6 to 8 h) using an appropriate endotracheal tube (Grade 2+).
R1.4 Within a standardised multimodal HAP prevention approach, we suggest not using the following methods to decrease ICU patient morbidity:
  Systematic early (< day 7) tracheotomy (except for specific indications),
  Anti-ulcer prophylaxis (except for specific indications),
  Post-pyloric enteral feeding (except for specific indications),
  Administration of probiotics and/or synbiotics,
  Early systematic change of the humidifier filter (except for specific manufacturer recommendations)
  Use of closed suctioning systems for endotracheal secretions,
  Use of antiseptic-coated intubation tubes or with tubes an “optimised” cuff shape,
  Selective oropharyngeal decontamination (SOD) with povidone-iodine,
  Use of prophylactic nebulised antibiotics,
  Daily skin decontamination using antiseptics (Grade 2−).
R1.5 In weaning of COPD patients from ventilation, we suggest using non-invasive ventilation to reduce length of invasive mechanical ventilation, incidence of HAP, morbidity and mortality (Grade 2+).

**Second area, DIAGNOSIS** What methods to diagnose HAP should be used to decrease ICU patient morbidity and mortality?

R2.1 We suggest not using the clinical scores (CPIS, modified CPIS) for diagnosing HAP (Grade 2−).
R2.2 We suggest collecting microbiological airway samples, regardless of type, before initiation of any change in antibiotic therapy (Grade 2+).
R2.2 Paediatrics We suggest collecting microbiological airway samples, regardless of type, before initiation of any change in antibiotic therapy (Grade 2+).
R2.3 We suggest not measuring plasma or alveolar levels of procalcitonin or soluble TREM-1 to diagnose HAP (Grade 2−).

**Third area, TREATMENT** What therapeutic options for HAP should be used to decrease ICU patient morbidity and mortality?

R3.1 We suggest immediately collecting samples and initiating antibiotic treatment taking into consideration risk factors for multidrug-resistant bacteria in patients with suspected HAP and haemodynamic or respiratory compromise (shock or acute respiratory distress syndrome) or frailty such as immunosuppression [95–100] (Grade 2+).
R3.2 We recommend treating HAP in mechanically ventilated immunocompetent patients empirically by a monotherapy, in the absence of risk factors for multidrug-resistant bacteria, non-fermenting Gram-negative bacilli and/or increased mortality (septic shock, organ failure) [101–113] (Grade 1+).
R3.3 The experts suggest not systematically directing empiric antibiotic therapy against methicillin-resistant *Staphylococcus aureus* in the treatment of HAP [114–119] (Experts Opinion).
R3.4 We suggest reducing the spectrum and preferring monotherapy for the antibiotic therapy of HAP after microbiological documentation, including for non-fermenting Gram-negative bacilli [114,115, 120–128] (Grade 2+).
R3.5 We recommend not prolonging for more than 7 days the antibiotic treatment for HAP, including for non-fermenting Gram-negative bacilli, apart from specific situations (immunosuppression, empyema, necrotising or abscessed pneumonia) [129–135] (Grade 1−).
R3.6 We suggest administering nebulised colimycine (sodium colistiméthate) and/or aminoglycosides in documented HAP due multidrug-resistant Gram-negative bacilli documented pneumonia established as sensitive to colimycin and/or aminoglycoside, when no other antibiotics can be used (based on the results of susceptibility testing) [136–152] (Grade 2+).
R3.7 We recommend not administering statins as adjuvant treatment for HAP [153–161] (Grade 1−).
